# Is Beauty in the Hand of the Writer? Influences of Aesthetic Preferences through Script Directions, Cultural, and Neurological Factors: A Literature Review

**DOI:** 10.3389/fpsyg.2017.01325

**Published:** 2017-08-03

**Authors:** Alexander G. Page, Chris McManus, Carmen P. González, Sobh Chahboun

**Affiliations:** ^1^Department of Teacher Education, The Norwegian University of Science and Technology Trondheim, Norway; ^2^Research Department of Clinical, Educational and Health Psychology, University College London London, United Kingdom; ^3^Interdisciplinary Centre for Science and Technology Studies, Bergischen Universität Wuppertal Wuppertal, Germany; ^4^Department of Language and Literature, Norwegian University of Science and Technology Trondheim, Norway

**Keywords:** script direction, aesthetic appreciation, perception, culture, beauty

## Abstract

Human experience surrounding the appreciation of beauty is not static. Many factors such as script direction and cultural differences directly impact whether, how and why we consider images beautiful. In an earlier study, Pérez González showed that 19th-century Iranian and Spanish professional photographers manifest lateral biases linked to reading writing direction in their compositions. The present paper aims to provide a general review on this topic and intends to highlight the most relevant studies reporting preferences in the appreciation of beauty in individuals with different reading and writing directions and belonging to different cultural backgrounds.

## Introduction

Beauty and the experience of the aesthetic are subjective and hard to define, as are their relationship to symmetry. Thinkers such as Aristotle thought of beauty as arising from symmetry, whereas Plotinus sought to uncouple this pairing (Anton, 1964). However, might not a portion of beauty rather stem from asymmetry? At the very least, we can say that the perception of an image as being aesthetically composed involves more than the beauty of the subject. The way the image is ordered, the placement of objects within it and their relationship to each other, all interact in an amalgam of composite elements vying for consideration. Although each ancient civilisation possessed its own artistic style, from which aesthetic preferences might be inferred, the earliest explicit ruminations on the subject stem from ancient Greece. While the various philosophers differ in their opinions, a common theme was that the appreciation of beauty could be measured in mathematical symmetry. Up to the twentieth century, architects and artists attempted to make their works more pleasing by using such mathematical constructs such as the Golden Ratio, and Pythagoras’ best-known evidence for the mathematical ordering of the cosmos revolves around music. However, in practice we see that neither painted portraits nor composed photographs tend to be symmetrical. In fact, there is a certain sterility to complete symmetry, which might decrease the aesthetic appreciation of the resulting image. Conversely, a certain degree of asymmetry lends dynamism to an image, which might increase appreciation (McManus, 2005).

One of the factors which affect the appreciation of an image is spatial ordering, as there are many dimensions that might be relevant to aesthetic appreciation (Gaffron, 1956). When that is said, the complex interplay of all these factors and planes would be exceedingly hard to research. Therefore, we shall concern ourselves here with directionality along the horizontal axis and the effects this can have on the response an image invokes in the beholder. This paper is a review of the literature dealing with the impact of directionality on aesthetics, the degree to which this is a result of cerebral processing, and the degree to which it results from habit or cultural variation. The most investigated manifestation of cultural difference in this regard is reading and writing direction (RWD), and we shall consider a multitude of studies comparing people whose writing systems have a right-to-left (R–L) directionality, and whose writing systems have a left-to-right (L–R) directionality.

The studies with which we shall concern ourselves are part of a range investigating preferences for image composition, although not all of these have expressed themselves in terms of aesthetics. However, the concept of aesthetics is so broad that it is fairly unproblematic to take these studies that speak of “preference” to concern aesthetics. More specifically, there is a range of studies suggesting that the lateral directionality of an image can influence the degree to which it is preferred (Levy, 1976; Beaumont, 1985; Christman and Pinger, 1997; Nachson et al., 1999; Chokron and De Agostini, 2000; Heath et al., 2005; Ishii et al., 2011). In a way, this places our work within the broader field of human universality. To what extent are our aesthetic standards innate to all people, and to what extent are they acquired expressions of cultural variation?

## Definitions of Aesthetics in Philosophy and Science

It is curious that, while many of these studies revolve centrally around the perception of beauty (e.g., Nachson et al., 1999; McManus, 2005; Masuda et al., 2008; De Agostini et al., 2010; Powell and Schirillo, 2011; Treiman and Allaith, 2013; McManus and Stöver, 2014; Chahboun et al., 2016), very few of them give any concrete definition as to what aesthetic entails. It is possible that this is a conscious choice, in the same way that Weber famously declined to offer a definition of religion in his treatise on the matter (Weber, 1922). There have been many theories concerning beauty and aesthetics, some taking them as essentially the same thing, others treating them separately. One model that distinguishes the two is the information-processing stage model, which views aesthetic experience in terms of its stages of cognitive processing (Leder et al., 2004; Leder and Nadal, 2014). These cognitive models for aesthetic processing generally involve the idea that images are more pleasing if they can be broken down into composite geometric shapes (Fell and Kopsiafti, 2016), although the experiments of McManus and Kitson (1995) did not support this. Another influential approach is derived from gestalt theory, and suggests that images are viewed holistically, being processed as an amalgamated whole of opposing forces (Arnheim, 1954, 1966), although McManus et al. (2011) found little to support this supposition. In general, aesthetic generalizations are easy to make but harder to test and so the generalizations often enter into the realm of ‘common knowledge’.

Throughout the ages, the best-known attempts to define such concepts as aesthetics and beauty have come from philosophy. As was intimated in the introduction, the earliest known explorations on beauty as a concept and what might define it stem from Greek antiquity, aesthetics itself being derived from the Greek word “Aisthētiké”, although the word in its modern usage traces back to Baumgarten’s *Aesthetika* of 1750. From antiquity onwards, many philosophers attempted to define the experience of the aesthetic, from seeing it as an emotional reaction to symmetry and harmony, to it being an expression of truth and knowledge through emotion rather than through reason (see Manns, 2016 for a longer account of the various philosophies of the aesthetic).

The views of philosophers on beauty are more striking with regard to their differences than their similarities. Even contemporaries, or near contemporaries, can have highly divergent impressions of what is beauty. One reason that beauty might be hard to define is the subjective and heterogeneous nature of the experience of something as aesthetically pleasing. Not only are there disagreements about what kind of experiences should be judged in terms of their aesthetic value, such as the ongoing debate as to what can be considered art, but the same stimulus can evoke wholly different reactions in each person who experiences it. In many ways, the subjective and emotional nature of aesthetic appreciation is the very antithesis to the detached reason and logic, which more typically comprises philosophy.

If we accept that the perception of beauty is largely an emotional response, we might be better served by moving away from accounts relying on consensus, such as that of Kant (1781/1914), and rather take on a more phenomenological viewpoint, such as that of Husserl (1900/1970). Phenomenology deals with the verity of experience without necessarily requiring a basis in objective reality. In this way, we can see the perception of a thing as pleasing based on the emotional responses of the viewer. Such a subjective, emotion-based view is reflected in the definition arrived at by Palmer et al. (2013).

“[T]he study of those mental processes that underlie disinterested evaluative experiences that are anchored at the positive end by feelings that would accompany verbal expressions such as ‘Oh wow! That’s wonderful! I love it!’ and at the negative end by ‘Oh yuck! That’s awful! I hate it!’ (Palmer et al., 2013:79)”.

Such a definition embraces rather than resolves the diffuse nature of perception, as it is based on the types of reaction an expression creates in the beholder, regardless of the fact that the types of phenomena that might provoke each reaction are endless and individual.

One way we might think of aesthetic appreciation is through the emotional response they produce. For instance, we might expect aesthetically neutral objects not to elicit any emotional reaction one way or the other concerning its level of beauty. The less pleasing an object is, the more negative emotion it will engender, whereas the more pleasing, the more positive emotion. The exact nature of this emotion is hard to mandate, and it will probably be different for each person. Thus, it seems wise of most studies not to rigidly define what aesthetic should be, but rather ask merely what is most pleasing, for whatever reason. This would also mean that those studies that do not mention aesthetics, but merely ask what is preferable to the participants, we can include as fitting our concept of constitutes aesthetically pleasing.

However, utilizing such a broad and encompassing view should not obfuscate the bases for the emotional reactions. From neuroscience and biology, we know that our emotional responses may be the embodied representations of deeper processes. We shall, in this paper, explore a number of theories for explaining aesthetic preferences, and even if one or more of them could be proved correct, this would not mean that the described mechanisms would be ones of which the individual is consciously aware. A person presented with two mirrored images of the same subject might just feel that one looks better or more right than the other, without being able to explain this sensation. For instance, Babel and McGuire (2015) point out that the experience of pleasure is dependent on the way an image is processed, an expression of fluency theory. This provides a straightforward mechanism to account for the effects of RWD on the aesthetic experience. Fluency theory refers to the ease with which an image is processed. The more fluently we perceive an artwork, the more positive will be our evaluation of it. When we speak of fluency with regards to artworks, several determinants should be considered. Reber et al. (2004) reviewed many perceptual variables that affect processing fluency, such as figural goodness, figure-ground contrast, stimulus repetition, symmetry, and prototypically, discussing how they might affect the experience of beauty. A related factor is how many times the spectator has seen the artwork. Repeated exposures mean more fluency and perceptual ease and, consequently, greater aesthetic evaluation (Cutting, 2006). The implicit acquisition of prototypes or grammars also influences perceptual fluency (Kinder et al., 2003). Finally, prior experience with the artistic domain, leading to a knowledge of the implicit structure of the image and specific processing expectations is also related to greater fluency and thus to better aesthetic judgements. As a conclusion, Reber et al. (2004) suggested that beauty is grounded in the processing experiences of the person viewing a stimulus.

It should be noted that these theories considering what is aesthetically pleasing do not require conscious awareness. Indeed, scholars such as Bateson (1972) have suggested that the coding and decoding of information needed for creating and understanding artistic expressions can only be done through the unconscious primary process. The unconscious nature of these processes means that their bases cannot be conclusively determined, although they might be inferred from research, which is part of our present purpose.

## Approaches to Directionality in Research

This review investigates the academic material on asymmetry in aesthetic appreciation. This entails a flow or an ordering of the elements in the work that may be regarded as directionality. Research has demonstrated preferential biases with regards to horizontal directionality, possibly related to the ordinary spatial bias known as pseudoneglect, which may be defined as asymmetry in spatial attention in neurologically normal individuals (Jewel and McCourt, 2000). However, while pseudoneglect appears to be a human universal, we must also consider other measures of directionality, which may be influenced by acquired factors, such as RWD. Throughout the available research, there are a number of different ways in which directionality in images is defined, some more straightforward, some less. Most studies utilize stimuli that they define as directional according to varying criteria. We might break these down into three main categories of directionality, i.e., direction of movement, implied direction of movement, and ordering of mass. The first of these is the most straightforward. As some studies use actual videos of motion, we have a fairly uncontroversial measure of the direction of movement (Nachson et al., 1999; Chokron and De Agostini, 2000; Maass et al., 2007; Ishii et al., 2011; Treiman and Allaith, 2013; Friedrich et al., 2014).

The second category is where movement is not shown, but rather implied. While a video of someone walking is an example of the first category, a still image of someone in the act of walking would be an example of the second. Examples of what would belong in this category would be still images of objects that have a clear front and back, such as people, animals, vehicles, etc. (Christman and Pinger, 1997; Nachson et al., 1999; Chokron and De Agostini, 2000; González, 2012; Friedrich et al., 2014; Chokron et al., 2016). Facing directions in portraits would be another example of this (Chahboun et al., 2016), as well as directionality in bodily composure, such as a subject leaning against a table or chair (González, 2012). Here also would be images of objects with an implied front, such as a triangle (Christman and Pinger, 1997) or objects that indicate a direction, such as a statue with an extended arm (Christman and Pinger, 1997; Chokron and De Agostini, 2000).

The last category is the least easily defined, and it is the spatial dispersal of objects, landscapes with points of focus, or the differential weighting of mass. Chokron and De Agostini (2000) utilize landscapes, as do many of the studies that draw inspiration from them (Christman and Pinger, 1997; Ishii et al., 2011; Friedrich et al., 2014; Friedrich and Elias, 2016). These create a directionality by including an object of focus, such as a beach scene containing a parasol. Weighting of mass could include the placement of objects or people of different size or height (Christman and Pinger, 1997; González, 2012; Chahboun et al., 2016).

We might also consider such elements here as location of agency, expressed for example in the differential distribution of people according to age and gender (Segel and Boroditsky, 2011). While this might be said to stretch the definition of directionality, we will see that similar processes are at work. González (2012) suggested that differential agency could also be a factor in images of a single individual as long as there were other elements within the image. For instance, if the person was standing next to an inanimate object, the person would possess greater agency than the object.

If we think about lateral biases in terms of neurological processing, it is well documented that pictorial stimuli are processed in the right hemisphere in right-handed people, and it is believed that this activation causes a leftward attentional bias (Blackburn and Schirillo, 2012). Therefore, Levy (1976) hypothesized that right handed people shift attention to the left side because processing an image is a function of the right hemisphere. This shift may also influence the impression of an image as more or less beautiful. Another author that believes in the importance of laterality is Beaumont (1985), who argues that the perception of a visual stimulus is biased more to the left visual field (right hemisphere) than the right visual field (left hemisphere) because most artists tend to produce their artworks putting the most important element to the right side and that is the reason we first perceive that element using the left visual field. These studies suggest that the neural organization of the cerebral hemispheres plays a role in the aesthetic preferences with regards to lateral organization (Levy, 1976; Beaumont, 1985).

All the available studies suggest that directionality can be defined in a number of ways, both physically and symbolically. This might be problematic, as it is entirely possible for an image to possess contradictory markers of directionality. If a person is depicted standing next to an object that is significantly larger, does the agency of the person or the weight of the object take precedence? What if that object is a pointing statue? This might seem academic, but it is a difficulty that arises all too easily. For instance, Liu et al. (2016) used an image of a caravel, a type of sailing ship, with a helmsman looking into the distance. The ship is located on one side of the page, and is sailing towards the other. This means that the directionality of the image could go either way depending on whether we consider ordering of mass or implied direction of movement as the most important criterion.

The definitions of directionality used by some scholars might also contradict those of others. Returning to definitions relying on differences in height, González’ (2012) uses Linear Ordering, where the directionality flows from the shortest to the tallest, for instance in images with siblings lined up from youngest to oldest. This operates on the logic that, sequentially, smaller amounts come before larger, and, chronologically, younger comes before older. However, Chahboun et al. (2016) define directionality through what they term the scanning hypothesis, which suggests that the gaze will be drawn to the taller individual first, then sweep over the others, meaning that directionality will flow from oldest to youngest. We might take this as a suggestion that scanning hypothesis also places distribution of mass over such symbolic aspects such as agency. If a man is depicted next to a woman, he will only be defined as the origin of directionality if he is taller than the woman is. If he is seated, for instance, the directionality will take her as its point of origin. However, this might also have a symbolic dimension, as height and size signify power and dominance (Dannenmaier and Thumin, 1964; Thomsen et al., 2011; Petersen et al., 2013). Differences in size and height are highly correlated with groups who differ in terms of power, such as men/women, and older children/younger children (Chahboun et al., 2016).

Apart from the aforementioned difficulties in determining the effect of directionality on aesthetic appreciation, we must also contend with the fact that so many other factors might affect preference. Recognizing this, many studies have tried to isolate the role or directionality by using mirrored sets of simple line drawings (Chokron and De Agostini, 2000; Ishii et al., 2011; Treiman and Allaith, 2013; Friedrich et al., 2014; Liu et al., 2016). Another study used profile drawings of people (Nachson et al., 1999), but the principle is much the same.

## Art and Image Composition

“De gustibus not est disputandum” goes the maxim, which in English reads “there is no accounting for taste”. The various criteria that are taken into account when determining aesthetic value are too many to accurately predict whether this thing or that thing is considered pleasing (see Palmer et al., 2013). For instance, we know that context determines preferences when selecting from a sequence, whether an extreme or the middle is more likely to be chosen (Bar-Hillel, 2015). When an artist creates a work of visual art, they have to decide on many aspects that will affect the artistic message. Some of these aspects are related to the spatial organization and orientation of the elements shown. McManus and Humphrey (1973) conducted a comparative study of the proportion of profile’s portraits and front ones in Western Europe. The researchers found that 1474 of 1500 portraits adopted a perspective of three quarters. Most interestingly, the proportion of them which were oriented to the left exceeded that expected by chance. Several authors consider that these decisions may be influenced by RWD, which is also related to the organization of the elements in the horizontal axis. RWD may affect how a person explores a page or a scene and focuses on certain aspects and not others. Therefore, the cultural variability regarding reading and writing systems may be an important factor that influence the way in which we perceive beauty, mentally represent it and finally express it (Gross and Bornstein, 1978). The only available study on the influence of RWD on lateral biases in art works is González (2012). She compared photographic portraits from cultures whose RWD is left-right (Spanish) and right-left (Iranian). Pérez González’s study uses a wide sample of pictures: 735 Iranian and 898 Spanish photographs from the Nineteenth century. There were five different types of composition: linear orderings (groups arranged by height), couples with both members standing, couples where one is standing and one sitting, individuals posing next to a table, and portraits. The results showed that there was a greater proportion of left-to-right photographs in the Spanish sample, in contrast to a greater proportion of right-to-left photographs in Iranian sample. Thus, script direction seems to affect the choice of compositional organization that artists make. Will it be able to affect also the aesthetic impression of the spectator?

There have been a range of studies investigating preferences as to image composition, although not all of these have expressed themselves in terms of aesthetics, but may have chosen the broader term “preference”. Returning to the study of McManus and Humphrey (1973), it was here argued that European portrait painters showed a preference for a slight rightward turn, so that the subject exposes the left cheek to the viewer. This is called a left-cheek bias, and while it is difficult to conclusively account for, there are hypotheses that might explain it.

According to Chahboun et al. (2016), there are two accounts for the left-cheek bias. The first of these is the emotionality account, proposed by Nicholls et al. (1999). The suggestion was that each side of the face conveys a different meaning, the left side carrying more emotional expression, the right being more serious. A tacit knowledge of this would determine what cheek was presented depending on what the intended message was. This might go some way to explain why the left-cheek bias is more prevalent in portraits of female subjects than male (McManus and Humphrey, 1973). The second explanation is the agency account proposed by Chatterjee (2002), and supported by Suitner and Maas (2007). Rather than any innate knowledge of emotionality, this relies on the embodiment of habitual action. The direction of reading, and the placement of the subject and object within the sentence, creates a predisposition that one side is more agentive than the other. This is supported by the fact that Maass and Russo (2003) found this tendency to be reversed in readers of Arabic, which is read from right to left. This tension between what is innate and what is acquired is one that will recur throughout this paper, and in particular we shall devote much space to the role of reading direction.

This idea that the left cheek bias might be due to an association between lateral directionality and agency is also supported when we view compositions of more than one subject. Maass et al. (2009) compared Italian and Arabic speakers, where both societies consider men more agentive than women, youth more agentive than old age. In images of groups, the people considered more agentive according to these criteria were placed on the left by the Italians and on the right by Arabs.

In this way, we can see that such tendencies in directionality are not isolated to portraits, but appear in artistic expressions generally. The ways in which directionality is expressed varies in these accounts with which we have so far dealt, as we have seen it expressed in both the bodily composure of the individual subject, as well as the placement of people within an image. González (2012) goes some way towards unifying these, by suggesting that relative agency will determine the placement of individuals within a group, but that when only one person appears, the directionality of bodily composure will take over.

## Sources of Directional Preferences

In the previous segment, we saw that art and other images are generally spatially arranged with a directional bias, that this bias was initially thought to be due to an innate human characteristic, and that this was then challenged. This is a recurring feature when we view the proposed sources of directional preferences. One lesson that should be drawn from this is to be wary when it comes to research with a Eurocentric leaning, and to focus more on cross-cultural perspectives. In the area, we are here investigating, this manifests in the new vistas of knowledge that open up once populations who read and write from right to left are taken into account (Friedrich and Elias, 2016).

In short, there is a range of studies that suggest a preference for pictures with the object of focus placed on the right, and other forms of rightward directionality. Studies of both animals and people have suggested that there is a perceptual and cognitive preference for left-to-right directionality (Friedrich et al., 2014). For instance, studies of aesthetic preferences have shown that people prefer artwork with the weight of the image on the right (Friedrich et al., 2014). Beaumont (1985) demonstrated a commonality of the way pictures are scanned, which probably accounts for this. People, it was found, viewed pictures from the bottom left, sweeping rightward and up. Here we might recall what was said earlier as to the subconscious processes that rule aesthetic judgement. Such scanning habits would have made images whose lines flowed in tandem with them more pleasant to look at, without the viewer necessarily knowing why.

According to De Agostini et al. (2010), there were initially two main explanations for the establishment of such scanning habits, both having to do with brain laterality. The first is that the object of focus is preferred when it is positioned on the right, as this would place it in the right visual field and consequently be processed by the left hemisphere. This would compensate for the otherwise disproportionate activation of the right (Levy, 1976; Goldstein, 2001). The other explanation is that the object of interest is so positioned so as to draw the eye to the rightward side of the image, thus placing the majority of the image in the left visual field (Beaumont, 1985). This would allow the lion’s share of the image to be processed by the right hemisphere.

However, it was eventually pointed out that these scanning habits need not be a result of neurobiology, but might simply be habitual action created by reading and writing. This is not an unreasonable supposition, as in literate people, reading and writing are highly practiced, to the degree of becoming automatic (Nachson et al., 1999). This hypothesis would be easy enough to test, as there is a range of writing systems which flow from right to left, such as Hebrew, Japanese and Arabic. If the previously documented L-R preference was due to brain lateralisation, it would remain regardless of RWD. If, however, it is a preference formed by reading habits, then we would expect to see a reversal.

The effect of habit can be more readily perceived in the study by Padakannaya et al. (2002). This was a comparison of unidirectional L-R readers, unidirectional R–L readers, bidirectional readers, and a group of illiterates from a R–L society. The bidirectional reading group were people with Urdu as their first language, but who had been schooled in English. The results were that the otherwise expected directional bias in the Urdu readers was weakened as a result of schooling in English, and was not present in the illiterates. Some ways in which an image can be asymmetrical are the posing directions, composition of objects, as well as lighting (Friedrich and Elias, 2016). When locating objects lit from different directions, L–R readers were quicker to identify objects lit from the upper-left, whereas R–L readers more easily located items lit from upper-right (Smith et al., 2015).

In fact, there are a number of ways in which RWD influence the way we function, and there seems no reason that scanning habits could not be one, a fact which sparked a range of research on the impact of reading and writing direction (RWD) on directional preferences. Because of this, while there are a great many factors in the composition of an image, the lateral organization, that is the directionality along the horizontal axis, and how it is affected by RWD, has been widely investigated (Nachson et al., 1999; Chokron and De Agostini, 2000).

An example of a study of directional preferences that takes neural organization as its explanation is that of Christman and Pinger (1997), although they acknowledge that the precise mechanisms were unclear. They investigated the preferences of an image over its mirror opposite, using three types of directionality, which were the placement of the larger object, the location of focal points and pointing. Their findings were that these directional cues affected the aesthetic appreciation of the images. While the results were not clear-cut, there was an overall preference for images with a left-to-right directionality. Another study is the one conducted by Nachson et al. (1999), who used profile drawings of people (which correspond to the category of objects with potential motion) and found similar results: L–R readers preferred L–R profiles, and R–L readers preferred R–L profiles (see also McLaughlin and Murphy, 1995).

Chokron and De Agostini (2000) was one of the early studies speculating on the relationship between RWD, scanning habits and aesthetic preferences. They compared 81 French and 81 Israeli subjects on their aesthetic appreciation on static images, moving images and landscapes. The results were a middle position, with the L–R readers showing the expected preference, but this being weakened or eliminated in the R–L group, rather than reversed. This suggested an interplay of causes, where brain laterality creates a base preference, which can be reinforced or counteracted by RWD. In fact, in the studies we shall be examining, it is notable that some phenomena are reversed due to RWD (e.g., Chahboun et al., 2016), whereas others show similar results as Chokron and De Agostini (2000), namely that we see a moderation rather than a reversal, again suggesting that some directional preferences are influenced by brain laterality, but not all.

Another oft-cited study is that of Ishii et al. (2011). They conducted a study similar to that of Chokron and De Agostini (2000), testing two groups with different reading directions on their preferences as to static line drawings, moving images and landscapes. Fifty English readers and the same number of Japanese readers took part. They found that the preferences as to static images and moving images follow reading direction. As for the landscapes, the Japanese readers showed a preference for landscapes with a rightward directionality, whereas English readers showed no preference. The authors themselves note the similarity of these findings with those of Chokron and De Agostini (2000), and state that their contribution was to show that the observed phenomenon extended to Japanese readers.

These studies suggest that readers of L–R and R–L scripts differ in their directional preferences when judging how aesthetically pleasing is a simple line drawing, with L–R readers showing a clear preference for drawings oriented rightwards, and R–L readers showing either a much reduced rightward bias, no bias, or the opposite leftward bias.

However, while Chokron and De Agostini’s (2000) findings has been replicated several times, the findings have not been consistent. Treiman and Allaith (2013) carried out a similar study, using mirrored static pictures and moving images, and also a much larger group of participants than had been the case in the other studies. Taking part were 736 Arab speakers (R–L) and 68 English speakers (L–R), and the result was that there were no difference was found in their preference for static pictures, and both groups showed a small predilection for the videos with a rightward directionality. They themselves point out that their pool of participants was much larger than that of previous studies, tacitly suggesting that their findings are more reliable. Such findings would seem to cast doubt on the supposition that reading direction causes directional biases, and that it might be down to neurobiological factors after all.

Noting the inconsistency of Treiman and Allaith (2013) with such earlier studies as Chokron and De Agostini (2000), Ishii et al. (2011), and Friedrich et al. (2014) repeated the experiments once again, as well as comparing the reception of static images to moving. They used 44 L–R participants and 40 R–L, testing them on 160 realistic images within four categories, these being mobile objects, landscapes, videos or mobile objects and videos of landscapes. All these were mirrored. L–R readers showed a preference for L–R directionality. R–L readers showed more mixed results, and did not show lateral preferences on all stimuli, although all preferences were stronger for the videos. They did find that in determining directional preferences in landscapes, direction of movement trumps location of object of focus.

Whatever mechanisms are at work in determining these lateral biases seem to be stronger with actual movement than with implied movement or distribution of mass. Viewing actual movement magnified the effect, revealing a markedly stronger bias than did still images. Even Treiman and Allaith (2013), who found no effect for line drawings, found the directional preference in moving images. Some of these studies have simply presented both images and asked which was preferred, but Maass et al. (2007) attempted to determine a more incremental degree of preference, as well as a more nuanced understanding of what “preference” might entail. They asked their participants to rate a series of film clips on their strength, speed and beauty. The participants consistently rated the videos higher on all scales in line with their RWD. This is an example of a study where the preferences follow RWD, evidenced by the complete reversal between the two groups.

Another attempt to resolve this controversy was conducted by Liu et al. (2016) replicated their research, which in turn was largely a recreation of that of Chokron and De Agostini (2000), but this time with Taiwanese. With 119 participants, they did not come near the numbers used by Ishii et al. (2011), but still enough to be statistically significant. Here RWD had a clear effect on aesthetic appreciation. In fact, while the traditional writing system of Taiwan has a R–L directionality, the Taiwanese are increasingly adopting L–R, for instance when writing on computers. Therefore, the authors expected this effect to be weaker than that observed among Hebrew speakers, but this was not the case.

Related to brain lateralization, handedness has been shown to be a factor (Levy, 1976; Freimuth and Wapner, 1979; Beaumont, 1985; Christman and Pinger, 1997; De Agostini and Chokron, 2002; De Agostini et al., 2010). The effects of handedness interact with those of RWD, and which is dominant shifts with age. This may be seen in children where the effect run counter. Their preferences are initially determined by their handedness, but eventually RWD take precedence, which is probably due to reading and writing becoming increasingly practiced over time (De Agostini et al., 2010).

Gender may also be a factor, as there is some suggestion that the L–R bias is stronger in males (De Agostini et al., 2010; Friedrich et al., 2014). This would also seem to have some bearing on the emotionality account of the left-cheek bias discussed above. Lateral preferences generally might also be said to have gender differences. Harris et al. (2009) investigated preferences as to how infants are held in images of the Madonna and Child. Women generally preferred images where the child was held on the left, whereas men showed no significant preferences, although the reasons for this were unclear.

Another possibility may be due to cultural factors. Nisbett (2003) outlined some differences between Asian and European thought, a difference that he suggests also affects aesthetic preferences. Some studies have suggested a salient influence from culture on people’s aesthetics and perception (Nisbett, 2003; Masuda et al., 2008). This is not surprising given that culture, after Bourdieu (1977), has been seen as a set of predispositions which might well affect pre-behavioral processes such as perception (Kastanakis and Voyer, 2014). However, does culture, beyond RWD, exert and influence on the preference of directionality?

When comparing people from societies with different writing systems, they are also likely to have significant cultural differences. Friedrich and Elias (2016) attempt to compensate for this by using two groups with different RWD, but who come from the same region in order to minimalize cultural distance. Their study compares the reception of mirrored image pairs and mobile images among groups whose language is Hindi (L–R) and Urdu (R–L). Their predictions was that they would have findings compatible with those of Prakash (2009), that the participants will display a leftward bias, but that this will be significantly weakened among the Urdu group. Further, they expected greater salience in the reactions to mobile images. These predictions were borne out, suggesting that cultural factors beyond habitual reading direction does not play a significant role.

## Complex Images

There are some studies that utilize more complex still images than simple line drawings. We already touched on this when dealing with the definition of directionality, but complex images such as photographs present particular difficulties, as they contain many elements that might suggest directionality, as well as many other aesthetical considerations (McManus et al., 2011; McManus and Stöver, 2014).

González (2012) who used 19th century studio photographs, comparing their reception among Spanish (L–R) and Iranian (R–L). There were five different types of composition, each with a differently defined directionality. It is therefore not surprising that the findings varied across categories. The reception of both the Linear orderings and Couples was fully explicable by RWD, whereas Chairs, Tables, and Portraits seem to have a base leftward bias, but which is affected by RWD.

Chahboun et al. (2016) investigated directional preferences with regards to professionally produced photographs. Spanish (L–R) and Moroccan (R–L) participants were presented with the photographs used by Pérez González, both in their original and mirrored forms. They found a general preference for photographs with a left to right directionality over those with rightwards directionality. However, these findings underscore the point we have made elsewhere in this paper that these lateral biases are subtle and easily overpowered by other considerations. This may be seen in the fact that this preference only revealed itself when their participants directly compared the mirror images, and even then, the effect was not particularly strong.

Complex images need not be photographs, however. We previously discussed Liu et al. (2016), whose first experiment was an attempt to resolve the controversy between Chokron and De Agostini (2000) and Ishii et al. (2011), using the same simple line drawings. However, they also conducted a second experiment, which used professionally designed images such as book covers, removing any text or other elements that could be used to distinguish the original from the mirror. They found that the effect that was found in their first experiment vanished. This suggests that the effect of laterality in determining aesthetic appreciation is relatively weak, and gets lost among other considerations within more complex images.

## Production Of Images

The previously viewed studies have focused on people’s preferences when responding to existing works. However, does this also transfer into the way people produce images? On the most basic level, this would appear to be the case. Lateral biases are attested in a variety of tasks that reveal the workings of basic level mechanisms of attention, perception, and action. RWD has indeed been shown to induce lateral spatial biases that affect how people draw (Vaid et al., 2002), visually explore (Abed, 1991), pay attention (Pérez et al., 2011) comprehend descriptions of events (Maass and Russo, 2003) and static scenes (Román et al., 2013), and how they mentally represent time (Ouellet et al., 2010) and numbers (Zebian, 2005). Most of the research on such biases have shown effects of handedness and brain lateralization, sometimes in the absence of effects of RWD. For example, on the most simple level, the participants used in the study by Singh et al. (2000) were asked to draw lines from both left to right and right to left using both hands in turn. The results were that, whichever hand was used, more accurate lines were produced when the participant drew in the direction which they would normally write.

When it comes to lateral biases, if we take the example of when people are asked to bisect a line, most of them place the dividing point slightly off centre, towards their direction of writing (Jewel and McCourt, 2000; McCourt, 2001; Fagard and Dahmen, 2010; Rinaldi et al., 2014). It should be mentioned here, however, that some people are more accurate in bisection than others, and that these also experience art as more evocative, suggesting that lateral biases affect the emotional impact of art (Drago et al., 2008). Similarly, when people are asked to draw a circle, reading direction will influence whether they do so clockwise or counter clockwise (Fagard and Dahmen, 2010). A number of studies also involve asking participants to produce more complex images in order to ascertain whether RWD affects the directionality of images (Alter, 1989; Singh et al., 2000; De Agostini and Chokron, 2002; Vaid et al., 2002).

With the production of more complex images, Jensen (1952a,b) found that right-handed children and adults of both sexes tend to draw profiles facing to the left side both in United States and Norway (L–R), Egypt (R–L), and Japan (top–down and R–L). In contrast, in left-handed children’s drawings the profiles works were oriented in both directions, with no particular preference. These results were later replicated by De Agostini and Chokron (2002). More complexly still, Vaid et al. (2002) asked their participants to draw various images, such as a tree, a hand and a fish, and it was found that both handedness and RWD could influence the directionality of the drawings. Presumably, this reflects the heterogeneity of left-handed people and shows that the orientation of the profile is not merely a function of how the hand holds a pen. Alter (1989) asked their participants to produce six sketches each, and found that handedness, gender and age affected the directionality in the produced images. Even having left-handed relatives was shown to influence the directionality of the drawings.

When it comes to visually representing causal flows, most societies do so in the same direction as the writing system. There are two hypotheses for this. Firstly, there is the matter of embodiment. We are so used to reading and writing in a given direction that we also habitually visualize processes running in the same direction. Consequently, extended practice in reading generates a directional schema for the flow of action along the lateral axis. In L–R readers, this schema places agents and causes on the left and patients and consequences on the right, generating a L–R spatial agency bias (Chatterjee et al., 1995). Secondly, there is the linguistic explanation. In 84% of languages, subject precedes object. This seems to be natural enough, as the subject, in a semantic sense, is the cause of the sentence’s action, whereas the object is that which is acted upon. As effect follows cause, so object follows subject. In this way, the “flow” of the action on the page will match the writing direction. However, what wold be the case for that minority of languages where object precedes subject? In a study by Maass et al. (2014) Italian, Arabic and Malagasy children were asked to draw interactions, such as an act of aggression. The result was that both Italian and Arabic children arranged the subject and the object of the action in the positions corresponding to their RWD. Malagasy children only did so intermittently, and this depended on the task, the motion of the scene and the salience of the word order. Thus, is the very concept of events in time manifested in a physical ordering of space.

Returning to lighting preferences, these might also be perceived in the production of images. It seems that images studied from a L–R reading society showed a preference for lighting from the upper left (Labar, 1973; Sun and Perona, 1998; McManus et al., 2004; Thomas et al., 2008), and given the perceptual findings of Smith et al. (2015), we can assume that this tendency would be weakened, or even reversed, in R–L reading artists.

## Conclusion and Future Directions

This paper was a review of the perception of aesthetic appreciation, with a focus on the effects created by script direction. One reason why such appreciation is so interesting may be because of its transcendent nature, which makes its various definitions subjective and therefore problematic. For this reason, we found it useful to acknowledge the philosophy of beauty, and yet choose a simple phenomenological understanding of aesthetic appreciation as an experience of preference (Nachson et al., 1999; Chahboun et al., 2016).

Alternatively, directionality was an easier factor to investigate, being based on more external, measurable factors (González, 2012; Treiman and Allaith, 2013; Chahboun et al., 2016). Studying directionality implies the study of a phenomenon that interacts with other phenomena, as was stated.

Most of the studies considered in this paper are heterogeneous and difficult to compare. Mainly because different methodologies have been used and individuals with various backgrounds have participated. We also acknowledged that some of the studies reviewed operated with different definitions of central concepts, such as directionality.

It should be clear from the various sources reviewed here that aesthetic preferences are complex, and are determined by a multitude of factors. While there are some apparent contradictions, we suggest that there is a sufficient degree of mutual corroboration and replication of results to conclude that lateral biases do exist. However, when more complex images were taken into account, we see indications that these biases are subtle enough to be overpowered when other factors come into play.

In this review, we highlight the importance of script direction in both the composition and the appreciation of artworks. We consider that this dual manifestation of brain laterality suggests that such effects would extend well beyond the field of aesthetic appreciation, and as such, there is fertile ground for further study.

With regard to the issue of human universality, our review echoes the earlier conclusions of Chokron and De Agostini (2000) that there does indeed seem to be a universal tendency for directional bias, but that this is prone to moderation by such factors as handedness and writing habits.

## Author Contributions

AP is the main author, and has written the bulk of the text. SC has written certain sections and has contributed with theoretical knowledge. CM and CG have read the various drafts of the paper, provided input as to the direction of the text.

## Conflict of Interest Statement

The authors declare that the research was conducted in the absence of any commercial or financial relationships that could be construed as a potential conflict of interest.
